# The experiences and beliefs of people with severe haemophilia and healthcare professionals on pain management, and their views of using exercise as an aspect of intervention: a qualitative study

**DOI:** 10.1080/09638288.2021.2018054

**Published:** 2021-12-24

**Authors:** P. McLaughlin, M. Hurley, P. Chowdary, D. Stephensen, K. Khair

**Affiliations:** a St George’s University of London and Kingston University, London, UK; b Katharine Dormandy Haemophilia and Thrombosis Centre, Royal Free Hospital, London, UK; c East Kent Hospitals University NHS Foundation Trust, Canterbury, UK; d Centre for Outcomes and Experience Research in Child Health, Illness and Disability (ORCHID) Research Unit, Great Ormond Street Hospital for Children NHS Trust, London, UK; e Haemnet, London, UK

**Keywords:** Haemophilia, pain management, reflexive thematic analysis, exercise, rehabilitation, rare disease

## Abstract

**Purpose:**

To explore the experiences, views and beliefs of people with severe haemophilia and healthcare professionals (HCPs) on approaches for pain management, as well as their views on exercise being used as an aspect of management.

**Methods:**

Taking a qualitative inquiry approach using focus groups and semi-structured interviews, participants included people with severe haemophilia living with chronic pain and haemophilia HCPs. Data were analysed using reflexive thematic analysis.

**Results:**

Fourteen men with haemophilia with a median age of 47 (range 23–73) and six haemophilia HCPs agreed to participate. Of the people with haemophilia, 11 attended two focus groups and three were interviewed over telephone. Healthcare professionals were interviewed face-to-face. Two themes were conceptualised from the data: (i) haemophilia management and pain management is discordant (imbalance between good haemophilia care but poor pain management, historical medico-social influences on pain management, the need for trust); (ii) uncertain about exercise but clear on what matters (conflicting views on exercise, the need for proof of safety, personalised care).

**Conclusions:**

Options for effective pain management remain limited and what is used is heavily influenced by beliefs and experience. Exercise as a treatment option in pain management is conceptually acceptable for people with haemophilia. Effective pain management requires understanding of individual beliefs and fears, and a personalised approach supported by knowledgeable, trusted clinicians.

**Implications for rehabilitation**
- Musculoskeletal joint pain and its relationship with bleeding in people with haemophilia continues to be a management challenge.
- Current pain management strategies are of limited effectiveness with little evidence of an approach that reflects the multi-modal pain experience.
- Whilst exercise and rehabilitation approaches are conceptually possible for people with severe haemophilia, barriers remain regarding perception of overall safety and effectiveness.
- People with severe haemophilia may consider exercise as part of a pain management strategy if it is individualised, and they are supported to do it by clinicians who understand them and their haemophilia.

## Introduction

Haemophilia is a rare congenital bleeding disorder characterised by a deficiency in circulating levels of clotting factor proteins VIII (haemophilia A) or IX (haemophilia B) [1]. The presence of adequate factor VIII and IX is central to the process of normal blood coagulation, enabling the generation of sufficient thrombin to stop bleeding and permit adequate healing to take place. In its untreated state, spontaneous bleeding into the muscle and synovial joints is the hallmark of severe haemophilia, with most children with severe haemophilia having their first bleed by the age of 4 years old [2].

Articular bleeding mainly affects the ankles, knees, and elbows, and over time the repeated exposure to blood products has a deleterious effect on the articular cartilage and bone health. Haemophilic arthropathy (HA) is the term given to this process and is characterised by bony deformity, cystic change, cartilage destruction, and pain [3]. For almost all people with severe and some with moderate haemophilia, current management is predominantly intravenous infusions of recombinant clotting factor proteins. This is done regularly at home to prevent bleeding occurring and is termed prophylaxis. For those with mild disease, and others who choose not to do “prophylaxis”, factor replacement is administered after a bleed has happened, known as “on-demand” treatment [4].

Chronic pain associated with the presence of HA is a significant comorbidity of haemophilia with figures suggesting between 20 and 68% of people with haemophilia are affected (PWH) [5,6]. Whilst there has been little in the way of research investigating pain mechanisms in PWH, it is widely assumed that similarly to osteoarthritis, acute and chronic nociception is the main mechanism [7,8]. However, there is growing awareness of the likely multifactorial influences on an individual’s pain experience whereby some PWH with no arthropathy report ongoing pain [9], as well the presence of pain in PWH not correlating with degree of joint damage and being a poor indicator of functional ability [10]. That said, PWH who are older and with a greater amount of joints affected by HA experience significantly more pain [11,12] and worse levels of psychosocial distress that negatively affect quality of life, mental well-being, function, and employment [13–15].

Despite the scale of the problem, there remains little in the way of published guidance for effective management of chronic arthropathic joint pain in PWH. Between 21 and 50% of PWH feel that their pain is poorly managed by healthcare teams [6,13] and this likely accounts, in part, for the wide range of strategies for pain management reported by PWH. Surveys in PWH have highlighted additional clotting factor treatment, opioids and non-steroidal anti-inflammatories, as well as rest, ice, compression, and elevation remain the most widely used methods for both acute and chronic pain [5,16], with other approaches including prayer, relaxation, deep breathing, and swimming also being used [17,18]. Whilst some have recently stated the importance of multi-professional expertise for pain management in PWH [19], psychology, physiotherapy, or exercise based strategies are seldom reported as being used by PWH [11,17].

The evidence base for effective and cohesive pain management approaches in PWH is lacking. NSAID’s and in particular COX-2 inhibitors, have shown more positive effect on reducing pain intensity than acetaminophen in those PWH who have HA [20,21], but worries remain about potential bleed risk if used for an extended time [22]. A psychologically based approach using an educational DVD that sought to influence self-efficacy in managing pain demonstrated shifting participants from a pre-contemplative to contemplative state of readiness to change [23]. Another small pilot study using hypnosis showed some positive, but not significant improvement effect on pain interference and quality of life [24] Whilst exercise in general appears safe to do with PWH [25], a recent systematic review found that there is low level evidence of effectiveness of many physiotherapy interventions (exercise, manual therapy, electrotherapeutic agents) on pain and functional outcomes [26]. A recent study implementing a combined intensive physiotherapy/occupational therapy intervention of strength and balance exercises, group work and education and rehabilitative approaches to activities of daily living was unable to show any significant improvements in pain or quality of life [27]. To the authors’ knowledge, there remains no published study that acknowledges or evaluates a multi-disciplinary approach to chronic pain management in PWH. Whilst some authors have identified fear of bleeding and further pain as barriers to being more active with haemophilia [28], there remains relatively little understanding of the lived experience of PWH and how they manage their pain, as well as the experiences of the HCPs who look after them. Insight into perspectives, thoughts and behaviours towards chronic pain from both parties is much needed if clinical care and individual approaches to pain management are to improve.

This study is part of a larger, ongoing research project that aims to develop and test the safety and acceptability of an exercise-based rehabilitation intervention for PWH who have chronic pain. Whilst it is acknowledged that the success of any pain management approach will likely require many individualised components, of which exercise may be one, the lack of quality research into the component parts requires an approach such as this to inform protocol development. A companion paper to this one explored how of a lifetime of painful experiences influenced beliefs about pain in adulthood in people with severe haemophilia [29]. The aim of the current study was to explore the experiences, views, and beliefs of PWH and haemophilia healthcare professionals (HCPs) around pain management strategies, and further investigate their views about exercise as a possible component in a pain management approach.

## Methodology

### Study design

As this was an explorative study seeking to better understand views and beliefs around pain and its management from PWH and haemophilia HCP’s, a qualitative approach using focus groups and semi-structured interviews was used. Focus groups bring together a group of people in order to discuss and share their own views and experiences around a particular topic, whilst semi-structured allow participants to speak freely and provide their own valid account around the topic at hand [30].

Whilst those in the research team working in haemophilia have long observed the complexities and difficulties in the management of chronic pain in this population, we acknowledge that approaches have been empirical at best, with varying degrees of success. There is an urgency in understanding the need to attempt to contextualise healthcare approaches to pain with the experience of the PWH receiving that care. As we were seeking to understand each participants own subjective reality about pain management and their views and beliefs about exercise, a relativist position with an interpretivist and phenomenological epistemology was taken. As no predetermined theories or frameworks were used, an inductive approach to analysis was used.

### Research team and reflexivity

The research team comprises the lead author who is a male clinical academic physiotherapist with extensive experience of working in haemophilia (15 years), a female nurse researcher with extensive experience in haemophilia, a male professor of rehabilitation science with an interest in pain management in arthritis, a male clinical academic physiotherapist specialising in haemophilia and a female professor of haematology. Regular meetings and supervision throughout this process helped ensure rigour and reflexivity was maintained.

### Recruitment

The study was advertised on social media and on posters that were displayed in haemophilia centres in south-east and north-west England. For those people with haemophilia, inclusion criteria were a diagnosis of severe haemophilia A or B, who self-identified as having persistent pain associated with their haemophilia, i.e., the presence of HA, aged 18 or over and with an absence of any other condition that would be responsible for the presence of persisting musculoskeletal pain. As the HCP’s most likely to have first-hand experience of pain in PWH, an invitation to participate email was sent via the professional clinical interest groups of haematologists, physiotherapists, nurses, and psychological professionals working in haemophilia in the UK. Inclusion criteria for the HCPs were the requirement to have experience in working clinically with PWH in adult haemophilia centres. All participants had to be able to communicate in spoken English. Those interested were encouraged to contact the lead author by phone or email to initiate further discussions to clarify any queries as well as check inclusion criteria.

### Setting/location

All interviews are focus groups were conducted between June 2019 and March 2020. Two face-to-face focus groups were held for PWH in south-east and north-west England. Due to Covid-19 restrictions, interviews with PWH were conducted over the telephone at a mutually convenient time agreed in advance with participants.

Although a focus group was planned for HCPs, meeting logistics agreeable to all who expressed an interest were not forthcoming, so it was decided to proceed with semi-structured face-to-face interviews instead. Interviews were arranged at times and places most suitable to the interviewee.

### Methods

Written informed consent was taken on arrival at the focus groups and face-to-face interviews and over email on the day of the telephone interviews. The study was approved by the St. Georges University of London Research Ethics committee (reference no. 2018.0309). The study was not pre-registered.

Topic guides were used for the PWH focus group, PWH interviews and HCP interviews based on the overall aims of the study (see Supplemental information). Developed in partnership with a person with haemophilia, they were informed by the current research literature in the area, clinical experience, and the research question at hand. Questions were open ended allowing naturalistic responses. Two moderators were present at the focus groups with one (the lead author) leading the group discussions and a second providing support in participant observation, making field notes and aiding those noted to be quieter to be drawn into conversations.

Both focus groups and all the interviews were digitally audio recorded and transcribed verbatim.

### Analysis

In keeping with the explorative nature of the study and with the intended focus on the subjective and sense making experience of pain management, an analytic approach using reflexive thematic analysis (RTA) was justified.

The key defining feature of RTA a compared to a coding reliability or codebook approach is that the codes and resultant themes are created at the intersection of the data itself, the analytic process and subjectivity [31]. Knowledge is produced with the researcher being acknowledged as an analytic resource [32]. RTA is described as a six phase recursive approach comprising (1) familiarisation with the data, (2) coding, (3) generating initial themes, (4) reviewing and developing themes, (5) refining, defining, and naming themes, and (6) writing up [33,34]. The lead author conducted the data analysis. Following familiarisation through immersion in the data, initial coding across both semantic and latent interpretations enabled initial theme development. As RTA is not a linear process, further refinement occurred as analysis developed, within and across each transcript dataset, moving backwards and forward through the stages.

*A priori* codes were not used to inform analytic approaches; therefore, the concept of data saturation was not used as it is incompatible with RTA as a researcher led, theoretically informed interpretive practice. The lead and last author discussed the data findings as initial coding led to theme development. The other members of the team were involved in iterative discussions to further refine the analysis findings as the final theme structure came to be constructed. All transcripts and other datasets such as fieldnotes were managed using NVivo 12 Pro^®^.

## Findings

A total of 14 PWH and six HCPs took part in this study. Of the 16 people who expressed an interest in attending the focus group, 11 PWH attended two focus groups due to availability on the day. After a second call for volunteers under the age of 30, and due to Covid-19 restrictions, three PWH were interviewed over the telephone. The HCPs were all interviewed face-to-face. The first focus group ran for 130 min, and the second for 180 min. The average interview length for PWH was 40 min (27–48 min) and 55 min (48–63 min) for HCP’s. Just over half of the haemophilia participants were known previously to the lead author as they attended the centre where he works. This familiarity was viewed as having a positive effect as it encouraged open and honest participation in the process, and participants felt safe and secure in the anonymity of their responses. There were approximately 15 h of recorded interviews transcribed.

The six HCPs included physiotherapists (*n* = 2), a haemophilia nurse (*n* = 1), haematologists (*n* = 2), and a psychology professional (*n* = 1), with an average of 12.5 years working in haemophilia (range 4–20 years). Table 1 presents the demographic information of the PWH who participated. Pseudonyms are included for use in the narrative that follows. Two themes were conceptualised from the data: (1) haemophilia management and pain management is discordant; (2) uncertain about exercise but clear on what matters. The subthemes within each theme are described in the text below.

**Table 1.** Participant demographics (pseudonyms) – people with haemophilia.

	Pseudonym	Age	Diagnosis	UK/non-UK born	Employment	Prophylaxis
1	Tony	55	SHA	Non	Y	Yes
2	Adam	28	SHA	UK	N	Yes
3	John	42	SHA	UK	Y	Yes
4	Jack	57	SHA	UK	N	Yes
5	Greg	39	SHB	UK	Y	Yes
6	Will	52	SHA	Non	N	Yes
7	Ivan	73	SHB	UK	Retired	Yes
8	Alex	58	SHA	UK	Retired	Yes
9	Owen	52	SHA	Non	Y	Yes
10	Andy	40	SHA	UK	Retired	Yes
11	Hugh	65	SHA inhibitor	Non	Y	Yes
12	Sean	23	SHA	Non	Student	Yes
13	Leon	28	SHB inhibitor	UK	Y	Yes
14	Nick	30	SHA	UK	Y	Yes

SHA: severe hemophilia A; SHB: severe haemophilia B; inhibitor: presence of antibodies that prevent factor replacement treatment from working effectively.

## Haemophilia management and pain management is discordant

Here, the assessment and management of pain, even as an acknowledged co-morbid aspect of life with haemophilia, is viewed as being less effective than haemophilia medical care, even though trust in the specialist healthcare team is high.

### Experience, knowledge, and understanding of pain

From an early age, PWH and their families have been conditioned to use pain as a marker to evaluate/diagnose bleeding so as to initiate a management strategy. The “if in doubt, treat” mantra was there to initiate clotting factor therapy as soon as possible to lessen bleed damage. The clinical language of possible danger was matched with behaviour of rest, subservience and waiting for resolution, but in adulthood and in an era of less bleeding, this behaviour is less than useful.

I think their go-to is, “This is a bleed,” and I actually think there’s quite a lot of undermanagement from MSK [services], because everything is put down to a bleed. (Rose, physiotherapist)

The reality that pain may exist without bleeding requires some form of acceptance internally but is not necessarily accompanied by a suitable solution, and so a newer developing form of interoception is required for those living or experiencing pain whilst on prophylaxis:

I was stuck in bed for a couple of… and it wasn't getting any better. And I was thinking it was a bleed, but it wasn't – it was tendonitis. (Jack, 57)

Whereas PWH have their own individual life story which feeds and moulds the narrative of their life lived with haemophilia, HCPs rely on the experience of hearing and seeing those living that life to build a picture of trying to understand what that must be like. PWH tend towards a biomechanical/biomedical basis for pain being present such as environmental provocations (walking on cobblestones), prolonged weight-bearing activity or bleeding. HCPs acknowledge these patient-reported “reasons” but hear and incorporate them into their own reasoning model, helping better understand observed patient behaviours:

I could see potentially how patients are self-diagnosing bleeds as a way of coming to terms or having a reason for their pain. And actually, if you’re ringing up work saying, “I can’t make it in today because I’ve had a bleed,” that’s quite different to, “I can’t come in today because my pain is too bad.” One is quite sort of acceptable and one is…. (Rose, physiotherapist)

### Care models and healthcare provision

A conflict appears to exist between PWH and HCP’s in how pain is managed. HCPs appear to be accepting of the fact that patients are not talking to them about pain because they talk about other profession specific issues (e.g., medications with the doctor), whereas PWH believe that they are being asked generic tick box questions:

The doctors, I'm getting “Are you taking your Celebrex? Are you taking your factor? Sorry you're in pain”. And that's where the conversation kind of ends, and you get fobbed off to the physiotherapist. (Will, 52)

Assessment of pain is challenging. PWH perceive that the value of pain rating or scoring scales is low and only helpful for clinicians.

I struggle with the 1 to 10 thing. Because, I mean, it’s just pain. It’s a different day. And I can’t… I mean, I can imagine a 10, but I don’t think I’ve had a 10. I can imagine a 10 because I’m a haemophiliac and I’ve had really, really bad bleeds. So, I struggle with the 1 to 10 thing. I generally just toss it at around 6 and leave it alone. It’s one of those questions that I don’t know how to answer. (Will, 52)

Clinicians recognise the inadequacies of historical care provision around pain in particular. Whilst confident in prescribing medication, they feel they have inadequate knowledge and skills to effectively manage chronic pain in its entirety.

I think I feel confident in asking the questions. I think how you deal with it is a real… can be a real challenge because, yes, okay, there are certain painkillers that I know how to prescribe, but I’m not a pain expert. (Ruth, Haematologist)

Even with concerns about their own skills and knowledge, clinicians see the need for approaches to pain management to improve so as to enhance care and be considered a normal and effective part of clinical review and intervention choice. PWH need validation of their life experiences in relation to their pain as they want to be part of a solution that works for them, and when it does it is beneficial and highly regarded.

I think one of the things I’ve noticed, particularly over the last couple of years, is you’re seeing doctors and physios kind of recognising that haemophilia doesn’t just occur in a bubble, the kind of text book methods, but actually you have the real life… you know, social life and things have to be fitted around that. And it’s been really nice to actually be given a bit more agency and responsibility to make decisions, and actively recognise that there is a lived context to what is possible. (Nick, 30)

### Trust in healthcare professionals

Feeling safe, being listened to and knowing that help in managing worries around living with haemophilia is available is important to PWH. Having the option of being able to call or drop into a centre is seen as a vital component of ongoing routine care as well as pain or bleeding that is not resolving as expected.

for me, it’s always been good to have the centre, where I can come and say, “Look, I’m not managing this. Something is wrong. I need help with it. (Tony, 55)

There is clearly a limited experience of pain management approaches that involve a haemophilia multi-disciplinary team intervention or approach. However, attending any other HCP who lacks knowledge of haemophilia is also regarded as ineffective. Barriers to accepting advice or intervention from non-specialists are ingrained in PWH and they are aware this is due to the rare nature of their disease as well as the uniqueness of their physical complaints associated with it.

I think I… I think haemophilia being reasonably quite a rare condition, I don’t think… Having grown up, I don’t have a natural trust of any and all clinicians to know why a joint might be the way it is, even if it’s quite a generic… even if the joint is damaged in a very generic way. (Leon, 28)

### Strategies for pain management

There is agreement with the HCPs and insight from those PWH that strategies employed for pain management are shaped by the life experience of growing up with haemophilia. Pain has always been associated with acute bleeding which can to some degree be managed by factor concentrate. For the most part everything that follows this initial thought process and decision making is linked to both the successes and failures of how individuals chose to manage these pain events.

Pain is danger. To me, pain means stop and treat yourself. And stop. (Hugh, 65)

Clinicians articulate that PWH coping and living better with pain in adulthood was linked to successes and acceptance of pain in their family and work life. Others reasoned that living with pain was more of a forced acceptance due to the limited unsuccessful options in current healthcare interventions:

From my side, it is… I think the patients that feel ground down by… it’s often too many things at the same time, which are overwhelming for them. And then all the sorts of things that they’ve done to manage their pain are no longer working, because they’re just too overloaded. (Mary, psychologist)

Using pain medication presents a dichotomy of opinion between PWH and clinicians. In an era of limited haemophilia treatment, opioid-based pain medications were standard. Older men with haemophilia have vivid distressing memories of becoming addicted and such addictions being almost ruinous to their life thereafter. Younger men have been socially conditioned to fear addiction from such medications and have as a rule resisted many attempts to take them as prescribed by clinicians even when they probably could be helpful.

I was under the impression that having… taking painkillers if you had pain, especially in my ankle, meant I might then put pressure on the ankle before was ready to have pressure on it because it wasn't hurting as much. So, I never did. (Greg, 39)

Co-infection with hepatitis C from contaminated blood products had also raised concerns about long term liver health with pain medications and becomes another considered reason why such medications are to be avoided. The avoidance of medication reflects a learned view that pain medications are risky and do not work for pain associated with haemophilia.

Options tried for pain relief exist on a continuum of good and bad consequence. Just to maintain daily activities and mobility PWH face a constant decision making process as to how far to push and challenge themselves within the realms of their daily pain – *‘So, it’s almost like it’s good, bad, but better. I think there is a lot to be said for keeping going sometimes, and just working through the pain to get to a better place.’ (Tony, 55)* Although others view such decisions as inevitably ending in failure:

… I actually am a bit eager and I have requested for physio/exercise referrals. I hope that the exercise would improve stuff, but what I’ve found is I jump on board each of these programmes with a bit of eagerness, I start trying to climb, and then I realise my joints aren’t allowing it, and then I… and then I give up. (Andy, 40)

## Uncertain about exercise but clear on what matters

When discussing exercise as a more specific component in managing pain, it was clear that most were uncertain of the rationale to do such a thing. Even with pain there is an acceptance by some, that exercise may provide some positive influence on their health and well-being; however, concerns over possible negative consequences based on previous experiences remain high. PWH identify function and less pain interference in day to day life as most important, and feeling supported to achieve this is essential.

### Barriers, enablers, and the need for more proof

Avoidance of bleeding and by default further pain, is by far the greatest barrier to being more active in daily life, and keeps PWH from taking the risk to do more.

… at the end of the day, people just want to live a pain-free, bleed-free life, and probably taking… people think that taking the more static… not doing something is less risky than doing something. (Leon, 28)

These concerns are recognised by clinicians as a hurdle for PWH in seeing a reason to do more with pain and for how HCPs can facilitate a way through this mind set. However, it also presents the clinicians with a dilemma in that they feel as yet unable to provide 100% assurance that such an approach is indeed safe.

I think the unknown, is what level of exercise is safe – and I don’t think we know that fully. I think we’ve got… all of us have got ideas, but I suspect there are clinicians who have got different ideas of what’s okay to do compared to others. We’ve got patients who do their own thing too. (Kate, physiotherapist)

Lack of motivation to change the current physical status quo exists with difficulty in conceptualising the benefit of doing exercise when in pain. Day to day activity that fulfils basic needs and requirements is seen as sufficient, and the idea of further physical challenge and exertion makes no sense. Whilst for others, being reassured by the efficacy of prophylaxis on bleeding enabled them to have confidence in “testing” their physical selves in specific exercise activities. This view is echoed by others who view an increase in pain as being less of an issue because its reassurance around bleed risk and after-effects that would encourage them to see exercise as an option:

… if I need to have a bit of pain and it doesn’t come with bleeding, to improve the condition of my joint, I would be more than happy to take that. I would take the pain knowing it’s not going to cause a bleed. (Andy, 40)

### Practicalities, logistics, and outcomes

Overall both PWH and HCPs were open and accepting of the premise of exercise as a component in pain management. For those that had previous positive experience with exercising with pain, there was a general acceptance that some more pain was acceptable in a longer term view of overall benefit.

PWH were clear that having someone they trust and who understood haemophilia and could understand their fears and anxieties was a key factor in how they would participate, as previous frustrations at non specialists had made them wary. Confidence in knowing they were being advised and shown the correct way to do exercises for example, helped moderate deep seated anxieties about further damage, risk of injury and being safe whilst exercising.

Anticipated personal outcomes were situated firmly in enhanced life participation and living well for PWH, and being able to function well despite their pain:

I’ve got… like the example of walking up the stairs to work, stuff like that, and just being able to… Feeling more confident to be… to kind of go out at night to some sort of bar or club and be standing up all night is a big quality of life improvement to me. (Leon, 28)

An acceptance of pain does not necessarily mean there would be no will to have it eradicated if possible, but there appears to be a realistic acceptance of what matters most in their day to lives.

I want to be kept going, I don’t want to be cured, if that makes any sense. (Hugh, 65)

## Discussion

The aim of this paper was to explore the experiences, views, and behaviours of PWH and HCPs about pain management and the use of exercise as a potential component of a management approach. Our analysis highlights that although PWH appreciate and value the expertise of the haemophilia care team, there is a view that they do not feel their pain is talked about or managed well within a haemophilia clinical environment. The current limited options for pain management for PWH remain fraught with fear of consequence and the need for reassurance as to what may be most suitable or effective for them.

Although PWH feel that issues relating to their pain are not captured in current haemophilia clinical setting, they also do not feel comfortable going elsewhere to other non-haemophilia specialists for pain advice, and so therein lies a conundrum. Haematologists and haemophilia centres are considered the primary port of call for pain management advice by PWH [6,11]. However, recent studies suggesting clinicians tend to underestimate pain in PWH [6,18] may help contextualise the reports of those PWH who feel their pain is poorly managed [13]. Our data suggest that such views could be explained in part by the fact that although HCP’s acknowledge the need to be able to manage pain within the haemophilia MDT, there is a perception that they do not have sufficient skills and knowledge to help PWH in their clinical care. This phenomenon has not been well documented to date, however, one qualitative study investigating haematologists experiences of managing PWH highlighted the difficulties faced with balancing being an expert in the disease area with the struggle of having to take on other multiple roles but without the necessary knowledge and skills [35]. It is clear that both clinicians and PWH need to find a way to work better together in managing chronic pain, to see people as more than their presenting condition and to identify care concerns and input that is meaningful to all [19].

The lack of a coherent approach to pain assessment in PWH that encompasses both physiological and psychological aspects has been highlighted [8]. For the most part routine, pain assessment has focussed on measuring intensity (VAS, pain rating scales) which PWH do find acceptable in acute bleeds [36]. Participants here perceive a low value to such rating scales for chronic pain, supporting the premise that it is not helpful in modern chronic pain management [37]. It is also reflective of other studies reporting that measuring intensity alone has limitations in PWH [38] and the use of such approaches misses the deeper lived experience and meaning of pain for an individual [39].

The recently updated haemophilia treatment guidelines from the World Federation of Haemophilia highlight pharmacological management as a first line strategy for both acute and chronic pain [40]. In particular, they recommend opioids as an option for chronic pain, whilst others have stated the need to avoid their use in PWH [19] and not to be commenced as a treatment option for those with chronic pain [37]. The participants in this study reported a dislike of pain medications and avoided taking them if at all possible. Perceived non-effectiveness and the dislike of how they made them feel were different to the reasons given in another qualitative investigating masculinity in haemophilia, where avoidance of pain medications was because taking them was viewed as an outward sign of their disorder [41]. Witkop et al. in their questionnaire study of PWH in the USA, noted only 28% took their pain medications as prescribed [42]. Whilst worries about toxicity and lack of effectiveness are common [43] a finding across multiple studies was that if people felt listened to, given appropriate information and were part of the decision making, then adherence and use of pain medications was better [43–45]. Healthcare professionals need to understand the life experiences of PWH to contextualise why they may not want to use pain medications and counsel them appropriately if they are felt to be of use in discrete situations.

‘No Aspirin, No Injections, No Exercise’ was a common sign seen above the hospital beds of PWH in the 1950 and 1960s, when treatment for active bleeding episodes was limited [46]. Exercise was synonymous with risk and danger of bleeding, and as a result many people went into adulthood with a view that being too active was risky. The narrative histories of those in this study, described in the companion paper to this [29], reflect these past experiences, which are also now being interwoven with trying to navigate and make sense of their chronic pain. The association between risk of musculoskeletal bleeding and physical activity perpetuates still in an era of improved haemostatic treatment. This historical reference point and the personal beliefs developed thereof for many PWH, sits rather awkwardly against a current biomedical paradigm that promotes physical activity for health benefit. Whereas other arthritises such as OA and RA have demonstrated self-management approaches using cardiovascular and strengthening exercise, education and coping skills to be useful adjuncts in pain management [47–50], it remains unclear if this is a safe and feasible approach for PWH. Unlike people with RA who expressed a fear of exercise making pain worse and causing more damage [51], the primary concern voiced here was the uncertainty and meaning of pain sensations. The fear is that being active with pain may provoke bleeding, as well as anxiety that the pain being experienced may actually be a small bleed that could be made worse with being active. This relationship between pain and bleeding has been explored by others who also noted activity avoidance, perhaps somewhat understandable in this context, was a key strategy in managing those fears [28,52,53]. Acknowledging and understanding the fears a PWH may have around their own pain sensations with exercise is important so as to formulate effective and mutually trustworthy interventions for pain management.

Chronic pain in PWH is more becoming more widely recognised as a moderator of physical activity, and by extension for some, planned exercise. Participants in our study were open to the possibility of trying exercise as a treatment option whilst having pain. However, they highlight that their reason to do so related more to being able to function better rather than cessation of their pain, which they acknowledge is unlikely to be “cured”. In a qualitative study of men with haemophilia, Taylor et al. [54] reported that their cohort had a very positive approach to exercise and PA and adopted positive coping strategies towards activity. This is in contrast to our study group who appeared more wary of being more active with exercise even though they could reason the benefits. This may be due in part to the participants in this study being recruited due to identifying as having chronic pain, whilst those in the Taylor study were noted to be positive towards PA and exercise as a group. One notable finding similar to ours however was the need to focus on what activities can be done (rather than what to avoid), with a similar focus on enjoyable non-traditional exercise activities (gardening, DIY, walking) being noted in another study by the same author [28].

An understanding of what is realistic, as well meaningful and potentially beneficial to PWH who have pain is important. Recent calls to action for pain management in PWH have highlighted the need for a multi-professional approach recognising the physical, social, and psychological aspects of care [19,55]; however, such an approach has yet to be investigated fully in this population. Current guidelines on pain management in arthritis from the European League Against Rheumatism (EULAR) recommend that a person centred care approach within a biopsychosocial framework, is delivered by a knowledgeable multi-professional team using a range of interventions that include exercise, social interventions, psychological support, joint specific treatment options, and sleep hygiene [56]. From our data presented here, we demonstrate that such an approach is appropriate, much needed and long overdue in the management of chronic pain in PWH.

We acknowledge that there were limitations to this study. This study was limited to people with severe haemophilia, as historically it is the condition most likely to present with painful multi-joint HA. Further investigations should explore if pain and concerns about bleeding influence decision making about pain management options in people with moderate and mild haemophilia. Another limitation may be the number and variety of HCP’s who took part in the interviews. Whilst it is possible that increased numbers of profession specific participants would have provided further data, time and resource available for to this study were limited. However as all professionals participating were expert clinicians in haemophilia care, working at five different haemophilia centres, and the fact that haemophilia care is delivered by a multi-disciplinary team, we feel that the views expressed represented a broad view of work practices and experiences.

Analytic process using a RTA approach rather than that of codebook approach to coding was employed here, and so may be considered a limitation in how reproducible the analysis of findings may be. This approach does not seek reproducibility of findings per se, but acknowledges the experience and influence of researcher background on interpretation. A key strength of this study was the condition specific and academic experience of the team. In a rare condition such as haemophilia, the need for this balance is important so as to drive forward quality research approaches that are also relevant to the people taking part in them.

Finally, although barriers and experiences about pain, exercise, and activity have been identified in this paper we do not in any way suggest that they may predict participation in rehabilitation activity. Further research needs to determine if any factors identified here can positively influence participation in a personalised, meaningful pain management programme, of which exercise may be a component.

## Conclusions

The need for improved, effective, and meaningful pain management strategies are much needed for PWH who live with longstanding chronic pain. Pain assessment remains perceived as low value, and is coupled with the fact that HCP’s in haemophilia feel ill-equipped to engage fully in the process. Even with this dissonance, there is substantial trust and well established therapeutic relationships between those with haemophilia and their healthcare team, providing an excellent foundation on which to build better pain management approaches. PWH want to feel reassured in doing activities that matter to them. It is imperative that pain management approaches are situated within an understanding of the individual’s lifetime of pain experiences, framed in the context of modern haemophilia treatments rather than historically inaccurate representations of haemostasis and designed around personally identified goals and functional aspirations.

## Supplementary Material

Supplemental Material

Supplemental Material
