# Supplementary material for: The experiences and beliefs of people with severe haemophilia and healthcare professionals on pain management, and their views of using exercise as an aspect of intervention: a qualitative study
Source: Disabil Rehabil. 2021 Dec 24;44(26):8420–8. doi: 10.1080/09638288.2021.2018054 (PMC12011021; doi:10.1080/09638288.2021.2018054)
Supplement: Supplemental Material [file IDRE_A_2018054_SM1435.docx]

**EXPLORING THE VIEWS AND OPINIONS OF HAEMOPHILIA HEALTHCARE PROFESSIONALS ON THE CURRENT AND POTENTIAL MANAGEMENT OF CHRONIC PAIN**

1. **Introduction**

- Introduce self and study
- Emphasise non-judgemental position, discussion not a Q&A
- Assure confidentiality
- Check permission to use audio recorder
- Mobiles off
- Thank all the participants for attending, no right/wrong answers, views and experiences are what matter

1. **General background information**

Invite all of the participants around the room to introduce themselves (include profession, years working in haemophilia and where they work).

1. **Clinician views of the lived (patient) experience of having chronic joint pain**

- Medical management of joint bleeding (before current medical treatments)
- Pain management in this acute phase
- Do you believe this affects pain experience in adulthood
- How does it relate with their haemophilia diagnosis
  - Do you have a different approach to severity of disease
- How it affects their day to day life
- Do you know/ask how pain affects a person’s work, study, family life
- Do you feel medical approaches have been helpful

(PROBE)

- Does it effect how active the patients are?
- How much? Why?
- Would you like to do more?
- What stops you?

1. **Methods of pain management being used**

- What current methods are they using
- What previously have they done
- Why did they choose these

(PROBE)

- Non medications such as illicit drug use, alcohol etc
- Did anything work well
- Why do they think it worked
- What did not work – why not
- Anyone else – GP?

1. **What is causing the pain**

- Why do you think people with haemophilia have pain

(PROBE)

-structural

-psychological

-mood

-expectation/normal

- Have you noticed any particular things that happen to make the people you look after have/report more pain
- What is the relationship between these events and their pain
- Do you think ‘pain’ can be made easier/managed better

1. **Using a rehabilitation intervention for joint pain in PWH**

- what do you think about using exercise and activity to help pain

- Have you ever tried?

- If – yes – how did it go?

- If - no – why not?

- what do you think may happen if you were to encourage this in an individual with a painful joint(s)

- Would you have the confidence in a clinical environment to recommend rehab

(PROBE)

- Do you think this may influence your current management approach?

- Why do you think it would be ?bad

- have any of your patients been advised not to exercise – by who?- Why?

- Why do you think something like rehab may be a good approach?

- Do you think it may affect your patients approach to working or daily life?

- What things are important for you to know if you were to encourage a patient to take part in something like this

(PROBE)

- Education

- how long (how many weeks)

- What do you think of exercising as part of a group

- what kind of things you would be doing

- where it would take place (is that important)

- opinion of haemophilia Dr’s – is that important

- Do you think it would be safe?

- Bleed risk

- more damage

- more pain

1. **Barriers and facilitators to a planned intervention**

- what is important for us to tell potential participants of a group such as this

- What things are important to help it run well

- Why do think people may not want to come

1. **Outcomes to measures**

We want to collect information about you before and after the intervention.

- What should we ask about to see if the group was helpful

- What matters most to you as clinician in trying to manage patient reported pain on a daily basis

1. **Anything else to add?**

Have you any questions or thoughts to add about the things we have discussed here today?

1. **Close the group/ Final thanks**
